# Can a Subjective Questionnaire Be Used as Brain-Computer Interface Performance Predictor?

**DOI:** 10.3389/fnhum.2018.00529

**Published:** 2019-01-23

**Authors:** Sébastien Rimbert, Nathalie Gayraud, Laurent Bougrain, Maureen Clerc, Stéphanie Fleck

**Affiliations:** ^1^Université de Lorraine, Inria, LORIA, Neurosys Team, Nancy, France; ^2^Université Côte d'Azur, Inria, Sophia-Antipolis Mditerrannée, Athena Team, Valbonne, France; ^3^Université de Lorraine, Perseus, Metz, France

**Keywords:** brain-computer interface, kinesthetic motor imagery, motor imagery questionnaire, BCI-illiterate, prediction of accuracy

## Abstract

Predicting a subject's ability to use a Brain Computer Interface (BCI) is one of the major issues in the BCI domain. Relevant applications of forecasting BCI performance include the ability to adapt the BCI to the needs and expectations of the user, assessing the efficiency of BCI use in stroke rehabilitation, and finally, homogenizing a research population. A limited number of recent studies have proposed the use of subjective questionnaires, such as the Motor Imagery Questionnaire Revised-Second Edition (MIQ-RS). However, further research is necessary to confirm the effectiveness of this type of subjective questionnaire as a BCI performance estimation tool. In this study we aim to answer the following questions: can the MIQ-RS be used to estimate the performance of an MI-based BCI? If not, can we identify different markers that could be used as performance estimators? To answer these questions, we recorded EEG signals from 35 healthy volunteers during BCI use. The subjects had previously completed the MIQ-RS questionnaire. We conducted an offline analysis to assess the correlation between the questionnaire scores related to Kinesthetic and Motor imagery tasks and the performances of four classification methods. Our results showed no significant correlation between BCI performance and the MIQ-RS scores. However, we reveal that BCI performance is correlated to habits and frequency of practicing manual activities.

## 1. Introduction

Brain-computer interfaces (BCI) allow end-users to interact with a system using modulation of brain activities which are partially observable in electroencephalographic (EEG) signals (Wolpaw and Wolpaw, 2012). A major modality of interaction is the detection of voluntary modulations in sensorimotor rhythms during Motor Imagery (MI). These sensorimotor rhythms are characterized, before and during an imagined movement, by a gradual decrease of power in—mainly—the mu-alpha (7–13 Hz) and beta (15–30 Hz) band and after the end of the motor imagery, by an increase of power in the beta band. These modulations are respectively known as Event-Related Desynchronization (ERD) and Event-Related Synchronization (ERS) or post-movement beta rebound (Pfurtscheller, 2003; Hashimoto and Ushiba, 2013; Kilavik et al., 2013; Lotte and Congedo, 2016). Two types of MI can be distinguished: Kinesthetic Motor Imageries (KMI) and Visual Motor Imageries (VMI). A KMI can be described as the ability to imagine performing a movement without executing it, by imagining haptic sensations felt during the real movement (i.e., tactile, proprioceptive, and kinesthetic) (Neuper et al., 2005; Guillot et al., 2009). In comparison, a VMI mainly relies on the visualization of the execution of that movement (Filgueiras et al., 2017; Mizuguchi et al., 2017). VMI and KMI share common neural networks particularly in the primary motor cortex, the motor cortex, the supplementary motor areas, the somatosensory cortex and the cerebellum, but also involve different cortical structures due to the intuitive nature of the KMI task (Filgueiras et al., 2017). More precisely, KMI produces a greater activation of the primary motor cortex and of the supplementary motor areas (Solodkin et al., 2004; Guillot et al., 2009). The resulting synaptic plasticity phenomenon makes the use of KMI-based BCIs, a promising instrument of acquisition and refinement of motor skills (Ridderinkhof and Brass, 2015). Moreover, KMI-based BCI use has shown encouraging results in the recovery of part of motor control for stroke patients (Cincotti et al., 2012).

Predicting the ability of a user to produce a MI remains a current challenge in the BCI domain (Jeunet et al., 2015; Ahn et al., 2018). The performance of MI-BCIs has been observed to vary across different users and different experiments (Ahn and Jun, 2015; Clerc et al., 2016). Moreover, 15 to 30% of users are not able to gain control of a BCI, a phenomenon sometimes called BCI illiteracy (Kübler et al., 2004; Allison and Neuper, 2010). Thus, a prediction tool would help determine the kind of training a subject will need to succeed in a KMI task (Mahmoudi and Erfanian, 2006). It would also allow researchers to readjust the BCI in order to keep the subject's motivation high (Lotte et al., 2013). Another interesting application is the introduction of a subject selection step prior to an experiment, for example to harmonize the population in a study with respect to BCI performance. More importantly, in the specific case of BCI-based rehabilitation, predicting KMI ability could support the development of tailored therapeutic KMI-based BCI protocols to help post-stroke patients to recover limb control (Braun et al., 2006; Butler and Page, 2006). In this particular case, the sensitivity of the BCI performance predictor is crucial. Indeed, a false negative will deprive a patient from the opportunity to use BCI-based care. Consequently, identifying whether a subject will perform well or not can save valuable time for all researchers while enhancing user experience.

Using a MI questionnaire as an ability predictor tool could be one possible way to estimate BCI performance. Indeed, in medical contexts, psychological assessment and questionnaires are probably the most accepted and validated methods to measure the MI ability of a subject (Vasylev et al., 2017). Nevertheless, in the BCI domain and to the best of our knowledge, only two studies have focused on predicting MI ability. These works studied two different MI questionnaires: the Kinesthetic and Visual Imagery Questionnaire (KVIQ) (Malouin et al., 2007; Vuckovic and Osuagwu, 2013) and the Motor Imagery Questionnaire Revised-Second Edition (MIQ-RS) (Marchesotti et al., 2016). The first study concludes that the KMI scores obtained from the KVIQ could predict the performance of a MI-based BCI for able-bodied subjects. The second study found that the representation of subjective behaviors, calculated using the MIQ-RS, and the control of the BCI seem to be strongly linked (Marchesotti et al., 2016). The performances in these studies (Vuckovic and Osuagwu, 2013; Marchesotti et al., 2016) were calculated for a classification task between right-hand versus left-hand MI tasks. However, commands and feedbacks are very different for hemiplegic stroke patients, since one of the hemispheres is affected by the stroke. Hence, with post-stoke patients, it seems preferable to discriminate MI from resting state. In healthy subjects, this discrimination can prove difficult as well, especially for subjects who have a poor lateralization profile (Rimbert et al., 2017). Therefore, studying these questionnaires as BCI performance predictors in an experimental condition involving MI vs. rest discrimination seems relevant. Finally, due to the small amount of studies, additional evidence is still needed before using the KVIQ-RS or MIQ-RS as a predictor of MI ability (and consequently, of BCI accuracy).

The goal of our study is to evaluate if the MIQ-RS could be a predictor of KMI-based BCI performance discriminating resting state versus right hand KMI. To verify this hypothesis, we recorded EEG signals from 35 healthy volunteers who had completed the MIQ-RS questionnaire prior to BCI use. We conduct several statistical tests to assess the correlation between the MIQ-RS questionnaire and the performances of four different classification methods. Finally, we propose to explore additional prediction markers, such as habits and frequency of practicing manual activities, to unveil significant correlations between these self-perceived factors related to everyday life activities and KMI-BCI accuracy.

## 2. Materials and Methods

### 2.1. Participants

Thirty-five right-handed healthy subjects (13 females; aged 25.83 years old; STD = 10.42) were recruited for this study. All the participants were novices in BCI and did not know what the MIQ-RS questionnaire was before staring the experiment. The participants had on average 3.29 years (STD = 3.06) of post-secondary education. This education level range ensured that all subjects were easily able to read and understand the written instructions of the MIQ-RS. More than providing a population comparable to the one involved in the previous studies, this avoided a possible bias linked to different help based on the needs of the subjects. The subjects had no medical history that could have influenced the task. The experiment followed the statements of the WMA declaration of Helsinki on ethical principles for medical research involving human subjects (World Medical, 2002). In addition, participants signed informed consent approved by the ethical committee of Inria (COERLE, approval number: 2016-011/01).

### 2.2. Questionnaires

#### 2.2.1. MIQ-RS

Prior to the experiment, the subjects were tested for their self-perception of VMI and KMI abilities via the French version of the MIQ-RS (Gregg et al., 2010; Loison et al., 2013). Concerning our choice to consider only the MIQ-RS, we note that it is more recent than the KVIQ (Gregg et al., 2010) and the results of Butler and Page (2006) indicate a that it shows similar internal consistency compared to the latter. Additionally, the MIQ-RS has been shown to be more reliable and valid for assessing MI ability in largest populations (i.e., both stroke and able-bodied populations) (Butler and Page, 2006; Gregg et al., 2010).

As described by Gregg et al. (2010), the MIQ-RS is a 14-item questionnaire that rates one's ability to imagine a movement. The questionnaire consists of seven visual and seven kinesthetic items. It requires 25 min to be administered. The tasks performed and imagined include functional and coarse movements. Each movement is described in detail and physically executed before being imagined, e.g., question 3: *Move your arm forward until it is directly in front of your body (still parallel to the ground). Keep your arm extended during the movement and make the movement slowly. Now move your arm back to the starting position, straight out to your side*. We refer the readers to Gregg et al. (2010) for the entire questionnaire. After imagining each movement, the participants use a seven-point Likert scale to rate the ease or difficulty of seeing or feeling the movement, depending on the instructions. A score of 1 means *very hard to see/feel* and a score of 7 means *very easy to see/feel*.

The total KMI (or VMI) score obtained by a subject corresponds to the average of the declared scores over the seven kinesthetic items (or of the seven visual ones) of the MIQ-RS, scaled from 1–7 to 0–100. It provides an easy-to-understand score from 0 (weak) to 100 (excellent). Note that it is an average score based on declarative answers of one's self-perception of the quality of motor imagery.

#### 2.2.2. Additional Information Survey

We also collected individual information through a small questionnaire to highlight our results from a user-centered point of view. Our hypothesis is that the manual activity rate of the subjects could impact their KMI ability. Therefore, in addition to their age, gender, and education level, we asked all the subjects to rate their self-perception of manual ability on a six-point Likert scale, indicating the frequency of manual activities, sport practice, and practice of a musical instrument in their everyday life (i.e., daily, weekly, monthly, annually, or never).

### 2.3. Experimental Task and Protocol

**Figure 1:**
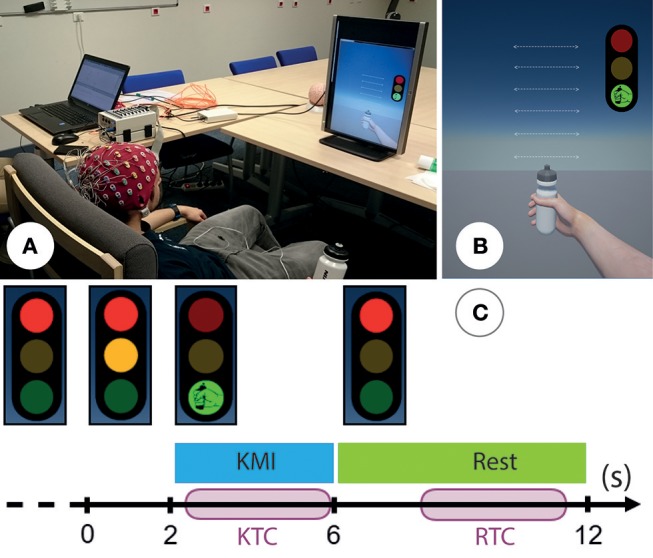
**(A)** Photo representing the experimental setup: subject is seated comfortably in front of a non-immersive virtual environment. Permission was obtained from the individual for the publication of this image. **(B)** The non-immersive virtual environment was composed of a three-color traffic light corresponding to the cues and a virtual right hand corresponding to the feedback. **(C)** Timing scheme for each trial: the subject performed right-hand KMI during 4 s when the light was green and was in a resting state when the light was red. An additional orange light warned the subject that the KMI would start soon. We segmented each trial into a kinesthetic time for classification (KTC) of 3.5 s during the KMI and a rest time for classification (RTC) during the resting state.

Each participant took part in one session of 80 min divided in 4 phases: (1) fill in the additional information survey and the MIQ-RS (25 min); (2) installation of the EEG cap (20 min); (3) one session of KMI during which participants had to perform one specific right-hand KMI task of grasping (15 min); (4) uninstallation and debriefing (20 min). During their KMI task, subjects were seated comfortably in front of a screen (Figure 1A) of a non-immersive virtual environment (ni-VR) composed of a three-color traffic light and a virtual right hand (Figure 1B). To support the generation of the KMI of grasping, we designed a Goal Oriented Imagery task (Vuckovic and Osuagwu, 2013). Hence, the subjects were invited to imagine clutching a bottle they had in their right hand as if they wanted to produce a water jet, while a similar bottle was also visible on the first-person view of the ni-VR (Figures 1A,B). The whole session consisted of one run with 40 trials. During each trial, they were invited to perform the KMI of grasping continuously during 4 s, as soon as the light turned green and while it remained so. The rest condition was similarly indicated by the red light (Figure 1C), lasting 6 s. Then, an orange light along with the red one, lasting 2 s, warned the subject that the KMI would start soon. This way the subject could be fully relaxed when the red light alone was on, in order to avoid motor preparation during the resting state. In summary, the subjects had to perform two distinct tasks during each trial: a right-hand KMI task (4 s) and a relaxation task (6 s). Between each trial we randomly allowed a time of around 2 s in order to prevent the subjects from anticipating the task.

### 2.4. Electrophysiological Recordings

EEG signals were recorded through the OpenViBE software platform (Renard et al., 2010) with a Biosemi Active Two 32-channel EEG system. The EEG was recorded from 32 sites in accordance with the international 10–20 system. The selected electrodes are *FC*_5_, *FC*_3_, *FC*_1_,*FC*_*z*_, *FC*_2_, *FC*_4_, *FC*_6_, *C*_5_,*C*_3_, *C*_1_, *C*_*z*_, *C*_2_, *C*_4_, *C*_6_, *CP*_5_, *CP*_3_, *CP*_1_, *CP*_*z*_, *CP*_2_, *CP*_4_, *CP*_6_, *P*_3_, *P*_1_, *P*_*z*_, *P*_2_, *P*_4_, P*O*_3_, P*O*_*z*_, P*O*_4_, *O*_1_, *O*_*z*_, and *O*_2_. These sites were localized around the primary motor cortex, the motor cortex, the somatosensory cortex and the occipital cortex, which allowed us to observe the physiological changes due to the kinesthetic motor imagery (Guillot et al., 2009; Filgueiras et al., 2017). Two additional electrodes are used in the Biosemi system: the Common Mode Sense (CMS) active electrode as reference and the Driven Right Leg (DRL) passive electrode, located over the parietal lobe and used to inject the current until the potential of the system and body are the same (Winter and Webster, 1983; Maby, 2016). An external electromyogram (EMG) electrode was added in order to verify that there was no movement during the KMI task. Impedance was kept below 10 kΩ for all electrodes to ensure that the background noise in the acquired signal was low.

### 2.5. Pre-processing and Classification

The recorded EEG signals were bandpassed using a 5th-order Butterworth filter between 8 and 30 Hz. Each trial was segmented into a kinesthetic time for classification (KTC) during the KMI and a rest time for classification (RTC) during the resting state, both lasting 3.5 s. The KTCs started 0.5 s after the go signal for the KMI activity (green light), while the RTCs started 2.5 s after the stop signal (red light) for the resting state (Figure 1C). For each session, we collected a total of 40 KTCs and 40 RTCs for a total of 80 trials for both classes. This number of trials is considered sufficient to achieve a classification and has been used in similar studies (Vuckovic and Osuagwu, 2013). We computed the performance of four different classification methods in a 4-fold cross-validation scheme. We proceed to detail the feature extraction and classification step of each method.

The first classifier, which we refer to as CSP+LDA, uses the Linear Discriminant Analysis (LDA) classification algorithm trained on features of the EEG signal, which was previously projected onto a lower-dimensional space. Let *C* denote the number of electrodes and *F* the new dimension of the projected EEG signal. We used a popular technique named Common Spatial Pattern (CSP) to reduce the dimension of the electrode space. During training, CSP yields spatial filters *W* ∈ ℝ^*F* × *C*^ which project the signal onto a surrogate space where the inter-class separability is maximized (Blankertz et al., 2008). The features we use to train the LDA classifier are hence the spatial log-variances of matrix *WX*_*i*_, where $X_{i}\in {\mathbb{R}}^{C\times T}$ denotes the *ith* trial. In this work we had *C* = 32 electrodes, *T* = 896 times samples, and we used *F* = 8 spatial filters (the first and last 4 filters generated by the solution of the CSP algorithm). We therefore yielded 40 8-dimensional feature vectors per class, for a total of 80 feature vectors.

The other three classifiers are Riemannian geometry-based classification methods, whose popularity has been rising in the BCI community due to their easy implementation and their enhanced performance (Lotte et al., 2018). Riemannian geometry-based methods work with the spatial covariance matrices of each trial, which live on the Riemannian manifold of symmetric positive definite matrices (Barachant et al., 2010). Hence, the features we used in the remaining three classification methods are the sample spatial covariance matrices $\Sigma_{i}\in {\mathbb{R}}^{C\times C}=\frac{1}{T-1}X_{i}X_{i}^{⊺}$ of each trial *X*_*i*_.

First, we used the Minimum Distance to Riemannian Mean algorithm (MDRM), which classifies each covariance matrix according to its Riemannian distance to the Riemannian mean of each class. This algorithm is detailed in Barachant et al. (2010). The second Riemannian algorithm is a modified version of MDRM, termed gfMDRM. The difference consists of applying geodesic filtering prior to training the MDRM algorithm using a method inspired by a generalization of the LDA algorithm to Riemannian manifolds (Fletcher and Joshi, 2004; Barachant et al., 2010). The last method, which we name TS+LR, is a technique that allowed us to project the feature space, that is the Riemannian manifold, onto a Euclidean space. To do so, we computed the Riemannian barycenter of the covariance matrices in the training set and projected the training and testing covariance matrices onto the tangent space at that point. Choosing the training set Riemannian mean as the projection point implies that the resulting tangent space is the best possible approximation of the original Riemannian space (Tuzel et al., 2008; Barachant et al., 2013). Then, since the tangent space is a Euclidean space, we trained and used a Linear Regression classifier.

### 2.6. Statistical Analysis

#### 2.6.1. Correlation of Individual Performances

A Shapiro-Wilk test of normality was performed, confirming normal distribution for kinesthetic, visual imagery scores, and classification accuracy (*p* < 0.05). In Table 1 we report the correlation between the performance of each classification method in terms of average classification accuracy (rows) and the average scores related to the KMI score and VMI score (columns) of the MIQ-RS (section 2.2.1) in terms of Pearson's correlation coefficient (r), along with the corresponding *p*-value. We also present the results of the same approach restricted to the KMI and VMI scores associated to the MIQ-RS questions involving hand movements in Table 2. Throughout this work, we adjust the significance rate α, which is originally assumed to be α = 0.05, using the Benjamini-Hochberg procedure for a false discovery rate (*q*-value) equal to 20% (Benjamini and Hochberg, 1995a). The correlations displayed in both tables remain not significant after this correction.

**Table 1:** Correlation between the performance (classification accuracy) of several classifiers and the kinesthetic and visuals scores of the questionnaire.

	**KMI scores (7)**		**VMI scores (7)**	
**Classifier**	***r***	***p*****-value**	***r***	***p*****-value**
MDM	0.097	0.579	−0.026	0.883
CSP+LDA	0.061	0.728	−0.161	0.355
gfMDRM	−0.081	0.644	−0.122	0.487
TS+LR	0.002	0.992	−0.176	0.311

*r denotes the Pearson's correlation coefficient*.

**Table 2:** Correlation between the performance (classification accuracy) of several classifiers and the kinesthetic and visual scores of the questionnaire for the questions related to hand movement.

	**KMI scores (3)**		**VMI scores (3)**	
**Classifier**	***r***	***p*****-value**	***r***	***p*****-value**
MDM	0.265	0.124	−0.057	0.746
CSP+LDA	0.233	0.179	−0.140	0.423
gfMDRM	0.171	0.327	−0.093	0.594
TS+LR	0.241	0.163	−0.166	0.340

*r denotes the Pearson correlation coefficient*.

#### 2.6.2. Group Performance Correlations

In order to study the correlation between the accuracy and both the KMI and VMI scores obtained using the MIQ-RS, we categorized our subjects according to two different criteria. Initially, following the bibliography in other perceived quality questionnaires, e.g., the SUS questionnaire (see Bangor et al., 2009), we considered that a KMI (or VMI) score is positive when it is equal to or greater than 70. We defined four categories of subjects: K+V+; K-V+; K+V-; and K-V-. K+V+ corresponds to subjects whose KMI and VMI scores are above 70; conversely for the K-V- category, where KMI and VMI scores are both below 69. In the K+V- category, KMI scores are above 70 and VMI scores below 69, whereas the opposite holds for K-V+. For each subject in each group, we considered their classification accuracy. To assess if the difference between the mean classification accuracy of a pair of groups is statistically significant, we performed an unequal variance unequal sample size *t*-test (also known as Welch's *t*-test) between all possible pairs of groups. In addition, we computed the effect size using Glass's Delta, using the largest sample as the control group when comparing between two groups, since the variances between groups cannot be assumed to be equal (Lalongo, 2016).

Then, we split our population into two groups according to their BCI accuracy. Subjects who performed higher than the group average were considered as strong performers and were therefore labeled “Perf+”; subjects with a weaker performance were labeled “Perf−.” Similarly, we performed Welch's *t*-test for the VMI score distributions and the KMI score distributions of the two groups (**Figure 3**).

Finally, to analyze the differences in BCI accuracy between subjects who practice a manual activity frequently (i.e., daily or weekly) and those who practice more occasionally (monthly, annually or never) we divided our population of subjects in two groups: “Manual+” and “Manual−” and performed Welch's *t*-test between the two groups.

#### 2.6.3. Time Frequency Analysis

For each group, we performed an event-related spectral perturbation (ERSP) analysis between 8 and 30 Hz with the EEGLAB toolbox (Delorme and Makeig, 2004). We used a 256 point sliding fast Fourier transform (FFT) window and we computed the mean ERSP 1s before KMI and 4 s during the KMI. ERSP allowed us to visualize event-related changes in the average power spectrum relative to a baseline (2 s) interval taken 2 s before each trial (Brunner et al., 2013). A permutation test for a significant level of 0.05 with a FDR correction using the EEGLAB toolbox was done to validate differences in terms of time-frequency of this ERSP (Benjamini and Hochberg, 1995b).

## 3. Results

### 3.1. MIQ-RS Score

The results of the MIQ-RS are composed of two scores: a KMI score and a VMI score, calculated according to the seven items which correspond to motor imagery, respectively kinesthetic and visual, performed by the subjects. On average, the KMI scores were lower than the VMI scores. The average KMI score was 67.75 (STD 13.06) while the average VMI score was 81.46 (STD 11.3).

### 3.2. BCI Accuracy

**Figure 2:**
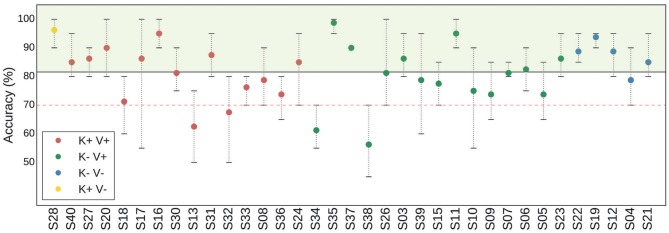
Figure denoting the average (point), minimum, and maximum (whiskers) classification accuracy per subject, computed in a 4-fold cross validation scheme. The red dashed horizontal line denotes the threshold under which a subject is considered BCI-illiterate. The black solid horizontal line denotes the average over all subjects. Four groups of subjects are identified with respect to their KMI and VMI scores: K+V+ (red); K-V+ (green); K+V- (blue); and K-V- (yellow). For example, K+V+ corresponds to the category of subjects for whom the quality of their KMI and of their VMI were rated over 70 points over 100.

The average classification accuracy between a right-hand KMI and a rest period was computed for 4 different classifiers (MDRM, CSP+LDA, gfMDRM, TS+LR, see Table 1). Throughout the rest of the paper, we report only the results that correspond to the classifier that produced the highest BCI accuracy, that is, the TS+LR classifier. The average accuracy of TS+LR was 81.57% (STD 10.06%); note that this classifier performed significantly better than the second best classifier (*p* < 0.001). Among the 35 subjects, 4 had an average accuracy that was below 70% (Figure 2).

### 3.3. Correlation Between MIQ-RS Scores and Classification Accuracy

No significant correlation was found between the KMI scores, the VMI scores and the BCI accuracy for any of the classifiers. We tested the correlation by considering all seven items contained in the questionnaire (Table 1). We also tested the three items (Table 2) that were closest to the KMI task of grasping performed by the subjects. No significant correlation was found for these three specific items either.

### 3.4. Correlation Between MIQ-RS Scores of Subgroups and Classification Accuracy

**Figure 3:**
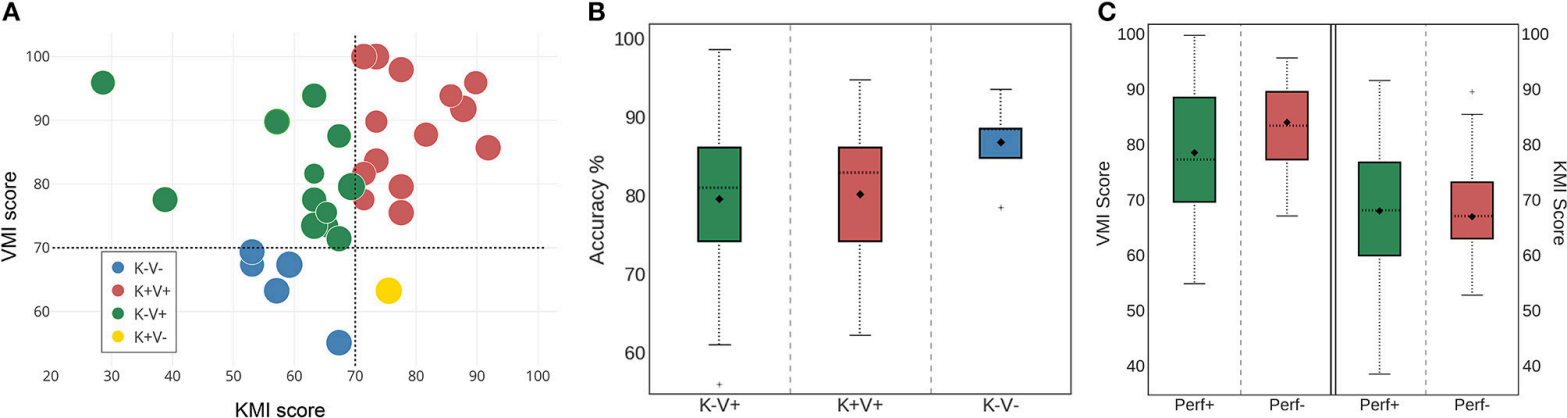
**(A)** Diagram representing the distributions of the subjects according to their KMI and VMI scores obtained from the MIQ-RS questionnaire. Disk diameter is proportional to good accuracy. **(B)** Boxplots showing the distribution of average classification accuracy for three groups: K-V+ (green—15 subjects), K+V+ (red—14 subjects), and K-V- (blue—5 subjects). Diamond markers represent the mean, while solid lines inside the boxes denote the median. The notches represent the confidence interval (CI) around the median. Notches are calculated using a Gaussian-based asymptotic approximation. K+V- group is not drawn because it has only one element. The separation is made with respect to the KMI and VMI scores of the subjects. **(C)** Boxplots showing the distribution of VMI scores (left) and KMI scores (right) for two groups according to classification accuracy: Perf+ (green—18 subjects) and Perf- (red—17 subjects).

Based on the MIQ-RS scores, the population of subjects was split into 4 subgroups as described in section 2.6.2 (i.e., K+V+; K+V-; K-V+; K-V-) (Figure 3A). The K+V+ subgroup is composed of 14 subjects (represented in red). The K+V- subgroup is composed of only one subject (represented in yellow). The K-V+ subgroup is composed of 15 subjects (represented in green). The K-V- subgroup is composed of 5 subjects (represented in blue). The reduced number of subjects in the group K+V- suggests that most subjects feel confident about their ability to visualize a task, whereas the low average kinesthetic score indicates how difficult it is for them to perform a KMI task.

The results of an unequal variance *t*-test showed no significant difference with respect to the classification accuracy for any pair of MIQ-RS subgroups (Figure 3B). The *p*-value between the K+V+ and K-V+ subgroups is equal to 0.87. In addition, since the sample size is small and does not allow us to assume that the data follows a normal distribution, we also performed a Mann-Whitney *U*-test, which does not show any statistical significance as well (*p* = 0.45) (Mann and Whithney, 1947). Note that the number of subjects in the K-V- is low and the resulting distribution of accuracy prevents us from drawing any conclusions about this particular subgroup. Finally, the effect size is also small for all pairs. It is equal to Δ = 0.0007 between the K+V+ and K-V+ groups; Δ = 0.0078 between the K-V- and K-V+ groups; and Δ = 0.0058 between the K+V+ and K-V- groups.

### 3.5. Correlation Between Classification Accuracy of Subgroups and KMI and VMI Scores

In line with Marchesotti et al. (2016), we categorized our population of subjects into two groups according to their BCI accuracy. Subjects with a performance score higher than the group average, which is equal to 81.57% (see Figure 2), were considered as strong performers and are labeled “Perf+”; subjects with a weaker performance are labeled “Perf−.” We found no statistical differences between the Perf+ and Perf− subjects comparing their KMI and VMI scores (Figure 3C). The *p*-value resulting from Welch's test is equal to 0.26, while the *p*-value resulting from Mann-Whitney's *U*-test is equal to 0.08 (significance level α = 0.05). Finally, the effect size between the two groups is equal to Δ = 0.34.

### 3.6. Correlation Between Individual Information and BCI User Accuracy Level

**Figure 4:**
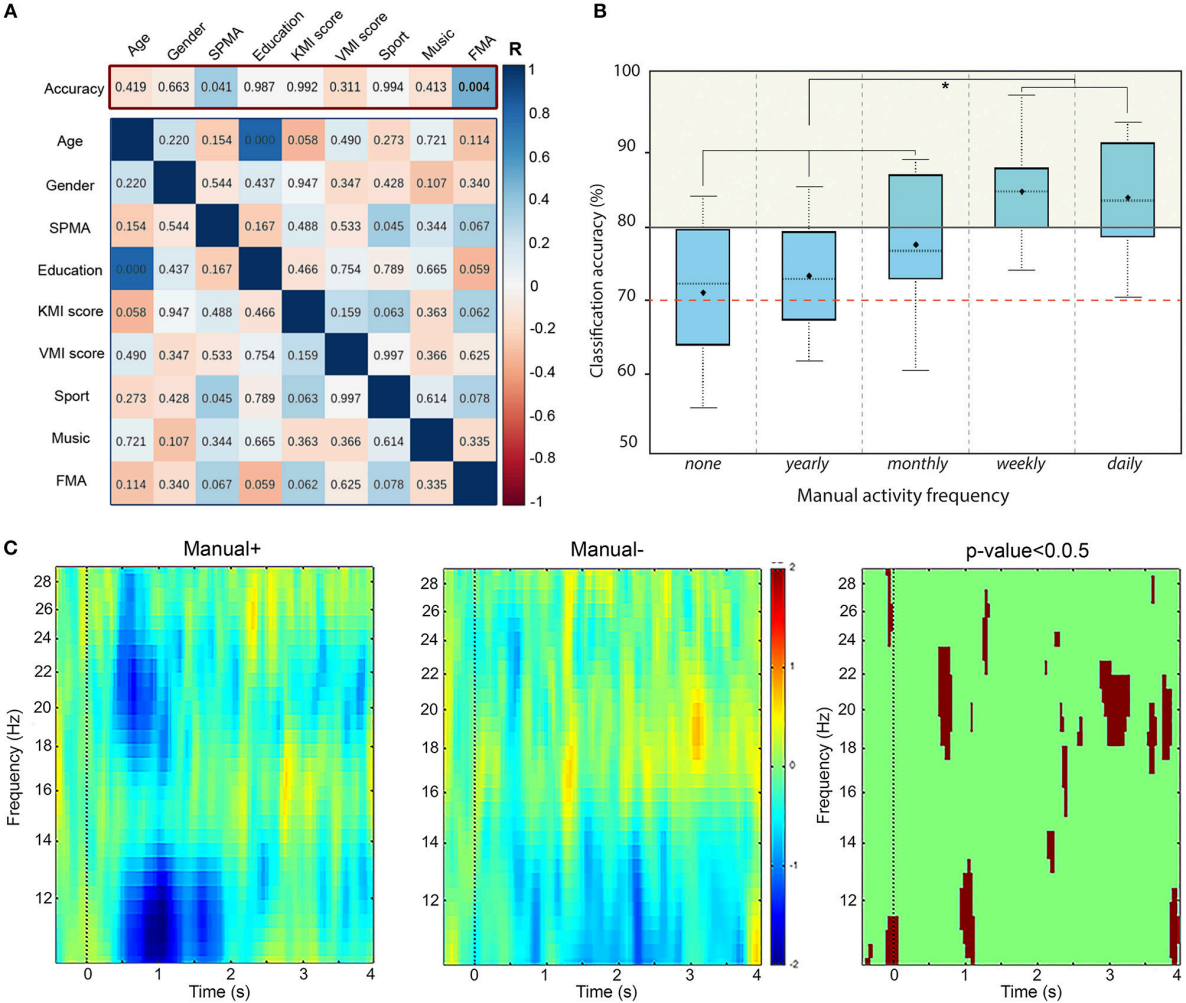
**(A)** Results of a Pearson correlation test between: (top row - primary hypothesis) the classification accuracy and individual factors; and (bottom matrix) the remaining pairs of scores. Colors indicate the r-score while numbers indicate the corresponding *p*-value. The significance level for our primary hypothesis is equal to α = 0.04 (adjusted for multiple comparisons). **(B)** Boxplot showing the distribution of accuracy for two groups according to the manual activity frequency: none, yearly, monthly, weekly, and daily. The red dashed line indicates the threshold for BCI-illiteracy. **(C)** Time-frequency grand average analysis (ERSP) for subjects who practice a manual activity with high frequency (Manual+) and subjects with lower frequency (Manual−) for electrode *C*_3_. A red color corresponds to an event-related synchronization (ERS) in the band of interest. A blue color corresponds to an event-related desynchronization (ERD) in the band of interest. Significant differences (*p* < 0.05) are shown in the final part of the figure.

Our primary hypothesis was the correlation between BCI accuracy and the collected personal factors (age, gender, education level, etc.). We computed correlation coefficients using two approaches. First, we calculated Pearson's correlation coefficients in order to be in accordance with the previous studies (Vuckovic and Osuagwu, 2013; Marchesotti et al., 2016). These results are displayed in the top of Figure 4A (red frame), where the colors correspond to the correlation coefficient and the numbers indicate the *p*-values. Then, considering the use of Likert scales in the KMI and VMI scores, we computed Spearman's correlation coefficients as well. The significance level α was adjusted for multiple comparisons using the Benjamini-Hochberg procedure in both cases.

In both tests, BCI accuracy is not significantly correlated to age, gender, education level, or declared frequencies of sport or musical instrument practice either (Figure 4A). Nevertheless, we can see a statistically significant positive linear correlation between BCI accuracy and declared frequency of manual activities -FMA- on Figure 4A, with *r* = 0.473, *p* = 0.004 and a significance level α = 0.04. The corresponding values for the Spearman test are ρ = 0.381, *p* = 0.024 and α = 0.04.

In addition, we investigated the existence of any correlation between the other factors as well. Note that no significant correlation is observed between KMI and VMI scores and any of the individual factors for either test. Figure 4A (blue frame, bottom) displays those results for the Pearson correlation coefficient.

### 3.7. Time-Frequency Analysis Based on Frequency of Manual Activities Profile

Motivated by the significant correlation between the BCI accuracy and the frequency of manual activities, we divided our population into two groups of subjects: those who declared to practice a manual activity frequently (daily or weekly) called “Manual+” and those who declared to practice less frequently (monthly, annually, or never) called “Manual−” (Figure 4B). Then, we performed a Time-Frequency analysis based on event-related spectral perturbations (ERSPs) for these two groups of subjects, illustrated in Figure 4C. In the beginning of the KMI task (0–2000 ms), an ERSP decrease (in blue) appears in the mu rhythm for both groups. However, the desynchronization is significantly stronger for Manual+ subjects (*p*-value < 0.05, corrected for multiple comparisons). In addition, the beta rhythm is more affected during the KMI for Manual+ subjects. Finally, an early beta rebound (at 3000 ms) seems to appear during the KMI task for Manual- subjects.

## 4. Discussion

This work shows that the performance of a BCI that discriminates between a right-hand KMI task and a rest state task is not correlated to the MIQ-RS scores. Our findings add nuance to the results obtained by previous studies (Vuckovic and Osuagwu, 2013; Marchesotti et al., 2016) and to the conclusion that the MIQ-RS could be used as a simple method to predict the user's performance in a MI-based BCI. In this discussion section, we consider which experimental conditions might explain these differences. We also discuss the observed significant correlation between the BCI classification accuracy and the declared frequency of manual activities (*r* = 0.473; ρ = 0.381; *p* < 0.04). These results open up new perspectives toward designing a specific questionnaire to predict the accuracy of a KMI-based BCI.

### 4.1. MIQ-RS KMI and VMI Scores vs. BCI Performance

In our article, we chose to assess individual correlations in terms of the Pearson correlation coefficient (see Tables 1,2). This choice was motivated by the results of two previous studies in this area (Vuckovic and Osuagwu, 2013; Marchesotti et al., 2016), which used the same correlation assessment method. Nevertheless the MIQ-RS questionnaire uses Lickert scales, therefore the correlation between classification scores and questionnaire scores might not have been linear. In order to have a more refined approach and confirm our initial results, whenever applicable, we computed the Spearman correlation coefficient as well. The resulting *p*-valuesproduced similar results, indicating no statistically significant correlations. In addition, we have tried to train several regression classifiers using the 3 or 5 items relevant with the KMI task contained in the MIQ-RS questionnaire. Five items of the MIQ-RS questionnaire concern arm movements and three items more specifically the right hand movements. The best result was given by the Elastic Net algorithm, with over 100 repetitions of 5-fold cross validation. However, our analysis showed no correlation with the accuracy for 3 or 5 items.

Several hypotheses could explain the absence of correlation between the classification accuracy and the MIQ-RS results. First, to limit experimental bias, the experimenter never helped the subjects understand the instructions of the MIQ-RS. The subjects, who were all novices in MI, may have met difficulties to conceptualize the nature of the mental task to perform; in particular (i) to perceive/feel/qualify what a KMI is and (ii) to produce a real KMI when faced with a complex sentence describing the task. Indeed, most of the tasks described in the MIQ-RS require a succession of gestures (e.g., *reach forward, grasp the glass and lift it slightly off the table. Now place it back on the table and replace your hand on your lap*) that can prove difficult to memorize. Subjects may have encountered cognitive (e.g., difficulty to figure out and/or to memorize the sequence of execution) or motivational (e.g., feeling of lack of confidence or of incompetence) barriers (Gregg et al., 2005). Note that the subjects could physically execute the expected movement only once. It is therefore uncertain whether they all have integrated it in a relevant manner to become able to mentally re-execute it, and even more, to be able to meta-analyze their self efficacy after the execution. Moreover, performing a KMI or a VMI immediately after movement execution might rely more on short term memory, which is not exactly the case for KMI-based BCI use. Second, it is also conceivable that some subjects met difficulties in estimating what their performance level was. Self-perception is a difficult parameter to qualify and rate. Indeed, taking into account self-perception theory (Bem, 1972), if a subject is novice in the analysis of their internal state, attitude, or self-capability, they do not have enough internal cues. Therefore, their self-interpretation might be weak and ambiguous. Bem indicated in this case that the subject acts as an external observer (Bem, 1972). This seems to be supported by the fact that the KMI scores of the MIQ-RS were lower than the VMI scores. Finally, we cannot exclude the possibility that some subjects have not answered in a rigorous manner.

### 4.2. Frequency of Manual Activity vs. BCI Performance

The ability to perform in KMI depends not only on the ability to mentally imagine the explicit elements of a movement (i.e., the conscious representation of the action to perform) but also on the ability to reactivate the implicit elements of this movement (i.e., unconscious aspects of a motor task such as all the feelings of haptic sensations) (Jeannerod, 1995). As observed in sport practices, physical ability, and mental imagery quality are closely related (Martin et al., 1999). Regular manual practice could indeed provide frequent cues and internal stimuli, enabling a subject to efficiently reactivate both of the KMI aspects. These parameters could therefore explain our results indicating that the subjects who are accustomed to manual activity were the most efficient. These results are also supported by the significant correlation between the subject's perception of their manual ability and their BCI performance. Another explanation could come from the fact that the physiological parameters of the contralateral motor area are influenced by the frequency of motor activity (Granert et al., 2011). Then, it is conceivable that subjects who have motor activities daily have a better physiological potential to perform well using KMI-based BCI.

### 4.3. Is Considering a Resting State Better for Control and Predictability?

Using left-hand KMI versus right-hand KMI is very common in the MI-BCI field. Nevertheless, we can question whether these two KMI tasks are most relevant for applications in this area, especially concerning KMI-based BCI performance estimation. A KMI generates an activity over specific regions of the primary motor cortex within the contralateral hemisphere of the body part used in the process (Pfurtscheller, 2001). Some BCIs are based on this contralateral activation to differentiate the cerebral activity generated by right-hand KMI from left-hand KMI. However, several studies have previously shown that some subjects have bilateral activity (Hashimoto and Ushiba, 2013; Rimbert et al., 2017). For such subjects, BCI performance would remain low for a classification task between left-hand KMI and right-hand KMI. Subsequently, the good accuracy obtained for all subjects in our study, as well as the low number of subjects that could be considered as BCI-illiterate in our study (i.e., only 4 subjects), may be linked to our classification task choice (right-hand KMI vs resting state). KMI is a complex task that requires specific skills, sometimes even adapted training (Jeunet et al., 2015, 2016). Moreover, performing KMI with the dominant hand is already not so easy for the subject. To include an additional KMI task involving the non-dominant hand maximizes that difficulty and could decrease BCI performance. This is not the case for the resting state, which is a more natural task. In addition, in the MIQ-RS questionnaire, the tasks to be performed by the subject are all directed toward the dominant hand. Finally, using a BCI based on right-hand and left-hand KMI to rehabilitate stroke patients is controversial, as one of the two hemispheres is often damaged.

Considering stroke patients, all these arguments merge into the fact that a right-hand vs resting state task would be more suitable to assess the relevance of MIQ-RS to predict the MI performance.

## 5. Conclusion

In this work, we answer the question as to whether the MIQ-RS can be used as a BCI performance predictor in condition other than right- vs. left-hand KMI tasks. We conducted KMI-based BCI experiments with 35 subjects that had completed the MIQ-RS. The classification task consisted of discriminating between a KMI task and a resting state. We then performed statistical tests to determine whether the MIQ-RS can be used as a BCI performance estimator.

Our results are twofold. First, we demonstrate that the MIQ-RS questionnaire cannot be used as a predictor of the KMI-BCI performance based on distinguishing between a resting state and a right-hand MI task. Consequently, the MIQ-RS should not be considered as a universal predictor of MI-based BCI performance. The lack of evidence that KMI-BCI accuracy is systematically correlated to the MIQ-RS scores should also raise awareness concerning the way the MIQ-RS might be used in motor rehabilitation protocols. Second, the significant correlation observed between BCI classification accuracy and regular practice of manual activity opens up new perspectives, both for future research targeted on BCI performance prediction and toward the design of user-centered MI-BCI. In particular, an example of a user-centered MI-BCI design is the inclusion of goal-oriented MI tasks, proposed to the subjects according to their daily manual tasks. Such designs can prove especially important in post-stroke rehab protocols. Overall, this study opens interesting research directions in human sciences (e.g., learning sciences, psychology), neurosciences and human-computer interaction.

## Author Contributions

SR, NG, LB, MC, and SF conceived and designed the experiments, performed the experiments, analyzed the data, contributed reagents, materials, and analysis tools, prepared figures and tables, authored or reviewed drafts of the paper, approved the final draft.

### Conflict of Interest Statement

The authors declare that the research was conducted in the absence of any commercial or financial relationships that could be construed as a potential conflict of interest. The reviewer BN and handling Editor declared their shared affiliation.
